# The impact of maternal and child health on sustainable development goals: evidence from Turkic Republics (2000–2020)

**DOI:** 10.1186/s12889-025-25172-z

**Published:** 2025-11-18

**Authors:** Gülay Ekinci

**Affiliations:** Istanbul Sabahattin Zaim University, Faculty of Health Sciences Health Management Department, Istanbul, Türkiye

**Keywords:** Econometric evaluation, Sustainable development, Turkic republics, Maternal mortality, Child mortality

## Abstract

**Background:**

The Sustainable Development Goals (SDGs) served as a global framework to promote health, well-being, and sustainable development. Among these, health indicators such as maternal mortality, under-five mortality, and neonatal mortality played a pivotal role in determining SDG performance, particularly in developing contexts such as the Turkic Republics.

**Aim:**

This research investigates, through empirical analysis, how major health-related indicators have shaped overall Sustainable Development Goal performance in six Turkic Republics—Azerbaijan, Kazakhstan, Kyrgyzstan, Tajikistan, Türkiye, and Uzbekistan—over the period 2000–2020.

**Methods:**

Using panel data techniques combined with econometric modeling, the study explored the dynamic interactions between selected health indicators and SDG scores. The independent variables comprised maternal mortality, under-five mortality, neonatal mortality, educational attainment, and health expenditure, while the dependent variable was the aggregate SDG score. Granger causality tests were applied to detect directional relationships, and variance decomposition (FEVD) was used to evaluate long-run equilibrium patterns and the explanatory contribution of each factor.

**Results:**

The findings demonstrate that maternal mortality (− 0.042) and under-five mortality (− 0.157) exerted significant negative effects on SDG scores, implying that elevated mortality rates hinder sustainable development progress. Educational attainment showed a significant positive coefficient (0.028), reinforcing its pivotal role in enhancing SDG achievements. By contrast, neonatal mortality and health expenditure exhibited no statistically significant influence on the SDG score. FEVD analysis further revealed that, by the tenth forecast period, maternal mortality accounted for 6.89% and under-five mortality for 8.65% of the forecast error variance in the SDG score, highlighting their persistent and measurable impact over time.

**Conclusions:**

The study underscores that social determinants—particularly health and education—are central to advancing sustainable development outcomes. Strengthening maternal and child health systems, alongside expanding access to quality education, emerges as a critical strategy for accelerating SDG progress across the region.

## Introduction

The Sustainable Development Goals offer an extensive global framework for recognizing and monitoring the most pressing challenges faced by humanity. Consisting of 17 overarching goals and more than 200 detailed targets, they encompass multiple dimensions of development. While each goal addresses a distinct thematic domain, the associated targets provide quantifiable benchmarks for assessing progress within that area. Among them, SDG 3 — Good Health and Well-being — is specifically devoted to safeguarding health and enhancing well-being across all stages of life. This goal incorporates essential health metrics, including maternal, newborn, and child health indicators, as well as chronic disease prevalence, immunization coverage, and life expectancy. Core targets for 2030 aim to reduce the global maternal mortality ratio to below 70 per 100,000 live births, cut neonatal mortality to under 12 per 1,000 live births, and decrease under-five mortality to fewer than 25 per 1,000 live births. However, persistent constraints in healthcare accessibility, particularly in low- and middle-income countries, remain a substantial barrier to meeting these objectives and strengthening the foundations of human capital [1].

Maternal and child mortality rates serve as pivotal markers of both the effectiveness of a country’s health system and its broader socio-economic conditions. Elevated mortality levels in these groups not only point to deficiencies in healthcare access and service quality but also reflect deeper structural challenges such as poverty, inequality, and limited educational opportunities [2]. These adverse health outcomes influence not only SDG 3 but also produce spillover effects on related goals, including Quality Education (SDG 4), Gender Equality (SDG 5), and No Poverty (SDG 1) [3].

For the Turkic Republics, exploring the links between maternal and child mortality and overall SDG performance is particularly valuable, as it can yield evidence-based insights for designing targeted health interventions with substantial development potential. In the absence of such focused inquiry, health policy may overlook critical mortality determinants, leading to inefficient allocation of resources and slower progress toward SDG milestones [4]. Furthermore, a nuanced understanding of these interconnections is essential for aligning national health priorities with the broader SDG framework, thereby fostering more integrated and sustainable policy outcomes.

While numerous global studies have examined the relationship between health outcomes and sustainable development, there is a notable lack of longitudinal, data-driven analyses focusing specifically on the Turkic Republics. Existing studies in this domain tend to adopt descriptive approaches, concentrate predominantly on macroeconomic variables, or generalize findings within broader regional groupings such as Central Asia or the post-Soviet space, often neglecting country-specific dynamics related to SDG 3 health indicators [5–7]. Moreover, quantitative investigations employing advanced econometric techniques—such as panel causality analyses and variance decomposition—to evaluate the impact of maternal and child mortality on SDG performance are notably limited, particularly in the context of countries transitioning from centrally planned to mixed-market economic systems.

The remainder of this paper is structured as follows. section. "Literature Review" provides a comprehensive review of the existing literature on maternal and child health within the broader context of sustainable development. section. "Data And Method" outlines the methodological framework, detailing the data sources, variable selection, and econometric techniques employed in the analysis. section. "Results" presents the empirical results, including findings from panel regression, Granger causality tests and variance decomposition. Finally, section. "Discussion And Recommendation" offers concluding remarks and discusses the policy implications derived from the study’s key findings.

## Literature review

The interconnection between sustainable development and health metrics has been extensively discussed in the literature related to human capital and development economics. According to Becker’s human capital theory (1964), an individual’s health substantially affects educational attainment, workforce productivity, and economic growth. Health thus acts as a key capital asset enhancing both personal well-being and economic performance. Specifically, maternal and child health outcomes influence the productive capacity of populations and thereby shape a nation’s development path [8, 9].

Development economics literature also emphasizes health in relation to its social determinants, including income, education, healthcare accessibility, and social support. High maternal and child mortality rates often stem from inadequacies in these determinants, worsening health outcomes and deepening inequalities [10, 11]. Elevated mortality often signals not just healthcare infrastructure shortcomings but also low educational engagement, limited economic and social participation of women, and persistent poverty. Such negative outcomes are not confined to SDG 3; they also exert influence on related goals, including SDG 4, SDG 5, and SDG 1 [12].

Empirical evidence demonstrates that elevated maternal and child mortality rates undermine sustainable development by constraining the formation of human capital, diminishing labor productivity, and slowing overall economic growth [12]. Maternal deaths, for example, can destabilize household structures, limit children’s educational opportunities, and perpetuate cycles of deprivation and inequality across generations [13]. As a result, nations with persistently high mortality rates encounter substantial obstacles in achieving the broader set of SDG targets.

According to the 2023 World Health Organization (WHO) report, the global maternal mortality rate has fallen by approximately 34% over the past two decades. While certain regions—such as Central and South Asia—have recorded notable improvements, significant disparities persist. Sub-Saharan Africa, for instance, still accounts for nearly 70% of global maternal deaths, largely due to shortages of trained healthcare personnel, insufficient medical facilities, and enduring socio-economic barriers [14, 15]. Studies from low- and middle-income countries consistently associate elevated maternal and child mortality with factors such as inadequate antenatal care, low rates of facility-based deliveries, limited immunization coverage, restricted decision-making power, and low levels of social capital [16–20]. In areas where socio-demographic indicators are weak, poor access to healthcare exacerbates risks linked to infections, postpartum hemorrhage, birth-related injuries, and shortages of essential medicines and vaccines [2, 21].

A considerable body of research also underscores the strong relationship between national income levels and the quality of maternal healthcare, with service standards in many low- and middle-income countries falling far behind those in wealthier nations [22–24]. Understanding the influence of maternal and child health metrics on SDG performance is therefore critical for identifying systemic weaknesses in healthcare provision and assessing overall developmental trajectories.

The Turkic Republics—Azerbaijan, Kazakhstan, Kyrgyzstan, Tajikistan, Türkiye, and Uzbekistan—provide a distinctive setting for examining these dynamics. Despite cultural and linguistic affinities, these nations differ considerably in socio-economic and political contexts. Following their independence in the early 1990 s after the dissolution of the Soviet Union, initial development strategies were heavily reliant on natural resource exploitation. However, limited economic diversification, political volatility, and other structural challenges have constrained long-term development [25–28]. Weak institutional reforms, entrenched authoritarian governance, the fragility of the middle class, and restricted democratic participation—compounded by the enduring geopolitical influence of Russia—have further slowed political and economic modernization. These factors present significant hurdles to achieving the SDGs in the region [29].

Although there have been improvements in health indicators, the Turkic Republics still face serious challenges in meeting SDG 3 objectives. Ongoing barriers include insufficient access to healthcare, a shortage of skilled birth attendants, and underdeveloped medical infrastructure, all of which contribute to persistently high maternal and child mortality rates. WHO statistics reveal considerable regional disparities within these countries in maternal, neonatal, and child mortality outcomes [30]. These inequalities not only constrain progress toward SDG 3 but also have adverse effects on broader development goals. Furthermore, a lack of comprehensive data and research on maternal and child health continues to impede the formulation of effective policies. Aligning national health strategies with SDG priorities and expanding evidence-based research capacity could significantly improve health outcomes and strengthen these countries’ contributions to global health progress.

### Purpose and significance of the study

The central aim of this research is to assess, through empirical analysis, how major health indicators relate to Sustainable Development Goal performance in the Turkic Republics over the years 2000–2020, using panel data methodologies. The investigation focuses on the influence of key health metrics—maternal mortality, neonatal mortality, and under-five mortality—on SDG 3, which is dedicated to advancing health and well-being. Moreover, the study incorporates structural factors such as educational attainment and health spending to evaluate their potential indirect roles in shaping sustainable development outcomes.

This study is significant in that it highlights health indicators not only as vital components of public health but also as strategic determinants of sustainable development. In the context of transition economies such as those of the Turkic Republics, reinforcing health systems is essential for improving social welfare, reducing inequalities, and achieving international development goals. Accordingly, the study seeks to provide evidence-based guidance to policymakers and contribute to health-oriented components of regional development strategies. Given the strategic importance of maternal and child health in the broader sustainable development context, the study addresses two core research questions:

i) Is there a statistically significant relationship between maternal and child mortality and SDG performance in the Turkic Republics? If so what is the direction and magnitude of this relationship?

This study tests the following hypotheses:

H_1_: Higher maternal mortality rates negatively affect the overall SDG scores in the Turkic Republics.

H_2_: Higher under-five mortality rates negatively impact SDG performance in the region.

H_3_: Increased education levels positively contribute to improving SDG scores.

H_4_: Neonatal mortality rates have a significant effect on the SDG scores.

H_5_: Health expenditures significantly influence the progress toward achieving SDG targets.

H_6_: There exists a long-term relationship between health indicators, education, and SDG outcomes.

This research fills an important gap in the existing literature by offering the first comparative panel data analysis of six Turkic countries from 2000 to 2020, directly linking maternal, neonatal, and under-five mortality to SDG performance. By applying a rigorous econometric framework—including OLS test, Unit root tests, Granger causality tests and variance decomposition—the study captures both short- and long-term dynamics. As such, it provides policy-relevant insights tailored to SDG-aligned health system reforms and contributes to the broader field of sustainable development policy in a region that has so far received limited empirical attention. By offering empirical evidence from a region characterized by shared historical legacies and contemporary development challenges, the study provides valuable insights for designing targeted health policies aligned with the SDG framework.

## Data and method

This study employs panel data analysis to investigate the relationship between selected health indicators and SDG performance. Although there are 26 recognized Turkic states and communities globally, this study focuses only on those countries with consistent, comparable, and long-term data availability. In this study, only the Turkic republics that provide regular and reliable international data on SDG monitoring (Azerbaijan, Kazakhstan, Kyrgyzstan, Tajikistan, Turkey, and Uzbekistan) were included, taking into account the reliability and comparability of the data for the period 2000–2020. Other Turkic countries were excluded due to missing or inconsistent data, which would have compromised the validity and robustness of the econometric analysis.

The dependent variable in the model was the SDG Index Score, obtained annually from the SDG Index and Dashboards Reports by Sachs et al. via sdgindex.org. This index serves as a comprehensive and standardized measure of each country’s overall SDG performance on a 0–100 scale. The primary explanatory variables were three key public health indicators: Maternal Mortality Rate, Neonatal Mortality Rate, Under-Five Mortality Rate. These mortality indicators were selected as they directly reflect progress toward SDG 3 (Good Health and Well-being) and serve as sensitive proxies for the quality and accessibility of health systems.

In this study, Gross Domestic Product (GDP), despite being a widely accepted indicator of economic development, was omitted from the analytical model to prevent redundancy. Instead, two proxy measures were employed to capture the economic and social dimensions typically associated with GDP: Health Expenditure (Hexp) and Secondary Education Enrollment (Edu). Health Expenditure reflects both the financial resources devoted to the healthcare sector and the broader economic capacity of a nation, while Secondary Education Enrollment represents the development of human capital and the strength of social infrastructure—both of which are closely connected to long-term economic growth. The choice of these proxies was guided by theoretical and methodological considerations. From a conceptual standpoint, health investment and educational attainment are integral to sustainable development and provide more targeted insights into the nexus between health and SDG performance. Methodologically, the inclusion of GDP alongside these variables could have introduced multicollinearity, thereby reducing the accuracy and reliability of the model’s parameter estimates. To address potential multicollinearity, especially between health expenditure and mortality variables, diagnostic checks such as Variance Inflation Factors (VIF) were conducted. The VIF scores ranged from 1.64 to 9.40, suggesting that multicollinearity was not a major issue in the model. Furthermore, tests for serial correlation (LM test) and heteroskedasticity confirmed the stability and reliability of the estimated model. As a result, the chosen control variables effectively represent the economic environment while maintaining robust and trustworthy estimation results. A detailed summary of variable definitions can be found in Table 1.

**Table 1:** Definition of variables

Variables	Definition	Unit	Abbreviation
SDG Index Score	The SDG Index Score in year t	Score Point (0–100)	SDGScore
Maternal Mortality	Maternal mortality rate in year t	per 100,000 live births	Matmort
Neonatal Mortality	Neonatal Mortality rate in year t	per 1,000 live births	Neonat
Under 5 years Mortality	Mortality rate, under-5 in year t	per 1,000 live births	U5Mort
Secondary Education	Secondary education completion rate is defined as the percentage of new entrants to the final grade of lower secondary education, regardless of age, relative to the population at the official age for that grade.	rate %	Edu
Health Expenditures	Current health expenditure per capita	Current US$	Hexp

Source: https://www.sdgindex.org; https://www.worldbank.org

The mathematical function of the model established in the research was as follows:

$$\begin{aligned}&\:SDGScore=f\\&\:[Matmort,\:Neonat,\:U5Mort,Edu,\:Hexp\:]\end{aligned}$$

The econometric model estimated within this equation framework was as follows:

$$\begin{aligned}\:{SDGScore}_{it}&={\beta\:}_{0}+{\beta\:}_{1}{Matmort}_{it}\\&+{\beta\:}_{2}{Neonat}_{it}+{\beta\:}_{3}{U5Mort}_{it}+\\&+{\beta\:}_{4}{Edu}_{it}+{\beta\:}_{5}{Hexp}_{it}{u}_{it}\:+{e}_{it}\end{aligned}$$

According to the model in the equation; “β_0_” coefficient constant represented the SDG score that was formed independently of the explanatory variables. “β_1_” for maternal mortality, “β_2_” for neonatal mortality, “β_3_” for u5Mortality, “β_4_” for Edu, “β_5_” for Hexp. The cross-sectional dimension of the panel data was expressed as “i” and the time dimension was expressed as “t”, $\:{u}_{it}$ captured unobserved country-specific effects, $\:{e}_{it}$ was the error term. “SDGScore” was defined as the dependent variable in the research.

This study applied a systematic econometric framework to analyze the proposed panel data model. The analysis began with estimating the model using Ordinary Least Squares (OLS) to gain preliminary insights into the relationships among variables and to assess the reliability of the estimated coefficients. The stationarity of the variables was then examined through multiple panel unit root tests, including Levin–Lin–Chu, Breitung, Im–Pesaran–Shin, ADF-Fisher, and PP-Fisher tests, which determined the integration order of the series and guided the choice of subsequent analyses. Optimal lag lengths were subsequently identified using information criteria such as LR, FPE, AIC, SC, and HQ, ensuring a balance between model parsimony and explanatory power. Given that all variables were stationary at level (I(0)), Panel Granger Causality Tests were conducted to explore short-run causal relationships among the variables. Lastly, Forecast Error Variance Decomposition (FEVD) was applied to measure the extent to which each explanatory variable accounts for variations in the SDG score, thereby revealing the nature of the system’s dynamic interrelationships. Taken together, these procedures enabled a thorough evaluation of both the short-run and long-run dynamics captured in the panel data. All statistical estimations and diagnostics were carried out using EViews version 10.

## Results

In this study, the findings were presented through a structured sequence of analyses. Initially, descriptive statistics and OLS regression were applied to provide preliminary insights into the relationships among the variables. Subsequently, panel unit root tests were carried out to verify the stationarity of the series, ensuring the reliability of the subsequent analyses. The optimal lag lengths were determined to capture the dynamic interactions within the model appropriately. Panel Granger Causality Tests were then conducted to identify the directions of short-run causal relationships, while Variance Decomposition analysis was performed to measure the contributions of each explanatory variable to variations in the SDG score. Through this approach, both immediate and enduring effects among the studied indicators were thoroughly assessed.

### Descriptive analysis

According to the descriptive information of the variables subject to the research;

- SDG score mean was 66.34 ± 4.01 (min:57.50, max:74.36)
- Maternal mortality mean was 37.57 ± 19.05 (min:12.55, max:86.86)
- Neonatal mortality mean was 16.32 ± 6.91 (min:4.65, max:34.67)
- u5mortality mean was 32.71 ± 16.27 (min:9.49, max:83.36)
- Education mean was 90.99 ± 8.09 (min:38.25, max:106.49)
- Health expenditures mean was 155.89 ± 149.68 (min:5.91, max:561.74)

**Fig. 1:**
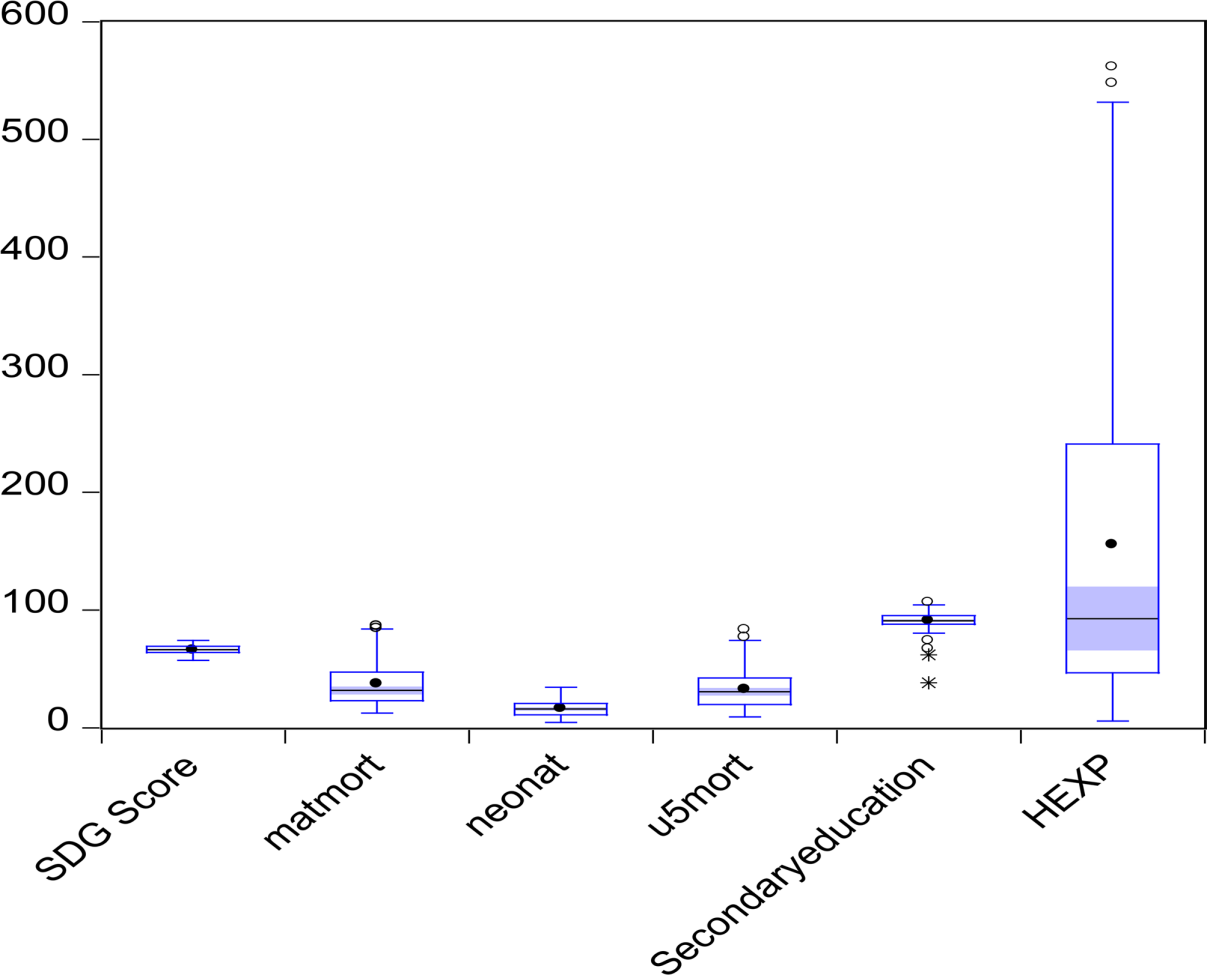
SDG scores and health indicators, Turkic Republics, 2000–2020

The relationship between the mortality values considered within the scope of the study and the SDG score was illustrated in Fig. 1. Descriptive analysis and the box plot graph summarized the central tendency and dispersion characteristics of the variables. The SDG score and neonatal mortality rate exhibited relatively low variance and a limited number of outliers, indicating a more homogeneous distribution. In contrast, the health expenditure variable displayed high variance and numerous outliers, pointing to significant disparities in healthcare spending across countries. Maternal mortality and under-five mortality rates also showed moderate dispersion and the presence of extreme values, reflecting inequalities in these health indicators. The education level variable, on the other hand, presented a more balanced distribution.

### Least squares test

In this study, a balanced panel dataset covering six countries over the period 2000–2020 was analyzed to examine the key determinants of SDGs. A fixed effects model was employed to control for unobserved, time-invariant heterogeneity across countries [31]. To address the presence of autocorrelation in the error terms, an AR(1) structure was incorporated into the model. The Hausman test strongly supported the fixed effects specification over the random effects alternative (*p* <.01), confirming the appropriateness of the chosen estimation strategy. Empirical results indicated that the u5mort exerts a statistically significant and negative influence on SDG scores (*p* <.05), highlighting the detrimental effect of child mortality on sustainable development outcomes. Similarly, matmort also had a negative impact, statistically significant at the 10% level. Secondary education enrollment rates were found to contribute positively to SDG performance, also significant at the 10% level. In contrast, the effects of neomort and health expenditures were statistically insignificant in this specification. The model demonstrated high explanatory power, with an adjusted R-squared of 0.97. Diagnostic tests for cross-sectional dependence—including the Breusch–Pagan LM, Pesaran scaled LM, bias-corrected LM, and Pesaran CD tests—failed to reject the null hypothesis, indicating no significant correlation in residuals across countries. The AR (1) coefficient was estimated at 0.64 with high statistical significance (*p* <.01), and the Durbin–Watson statistic of 2.15 suggests that autocorrelation was effectively corrected. Additionally, Variance Inflation Factors ranging between 1.64 and 9.40 confirm the absence of problematic multicollinearity among the explanatory variables. Overall, the model satisfied key econometric assumptions, and the coefficient estimates were robust and interpretable. The findings underscored the critical role of improving maternal and child health outcomes, as well as enhancing access to education, in advancing sustainable development performance across countries (Table 2).

**Table 2:** Least square test results

Dependent Variable	Independent Variables	Coefficient	Prob.
SDG score	Matmort	−0.042305	0.0707***
	Neonat	−0.089791	0.4751
	U5Mort	−0.156656	0.0207**
	Edu	0.027999	0.0764***
	Hexp	−0.002113	0.3307
	AR(1)	0.640978	0.0000*
	C	72.25269	0.0000*

Source: Prepared by the author

Hausman Tests:0.0000; Breusch-Pagan LM:0.6098; Pesaran scaled LM:0.7018; Bias-corrected scaled LM: 0.5886; Peseran CD Test:0.0784; Variance Inflation Factors: 1.64–9.40; R:0.96; Adjusted R^2^::0.97; *,**,***significance at %1,%5,%10 level.respectively

### VAR lag order selection criteria and unit root test results

To examine the causal relationships among the variables, the optimal lag length for the Vector Autoregression (VAR) model was determined using several information criteria. Table 3 presents the selection statistics, including the Likelihood Ratio (LR), Final Prediction Error (FPE), Akaike Information Criterion (AIC), Schwarz Criterion (SC**)**, and Hannan-Quinn Criterion (HQ). Among these, the LR, FPE, and AIC criteria all suggest an optimal lag length of four (4). The SC and HQ criteria indicated lag length of three (3). Based on the literature AIC and HQ are widely recognized for providing more accurate model specifications without overfitting, and therefore, the lag length of 3 was selected for the VAR model [31, 32]. This choice ensures that the model captures the necessary dynamics of the data while avoiding the problem of over-parameterization. Furthermore, using this lag structure satisfies the stability conditions of the VAR model and enhances the reliability of subsequent causality tests (Table 3).

**Table 3:** Lag length criteria test results

VAR Lag Order Selection Criteria						
Lag	LogL	LR	FPE	AIC	SC	HQ
0	−1873.466	NA	5.54e + 10	41.76592	41.93257	41.83312
1	−924.5630	1750.200	85.95322	21.47918	22.64575	21.94961
2	−707.6439	371.1727	1.558460	17.45875	19.62525	18.33241
3	−607.1001	158.6357	0.381311	16.02445	19.19087*	17.30134*
4	−561.5853	65.74364*	0.324873*	15.81301*	19.97936	17.49312
5	−542.4390	25.10291	0.515023	16.18753	21.35381	18.27088
6	−499.5419	50.52320	0.505120	16.03427	22.20046	18.52084

Source: Prepared by the author

The stationarity levels of the variables used in the study were assessed using various unit root tests (Levin, Lin & Chu, Breitung, Im-Pesaran-Shin, ADF, PP). The test results indicated that some variables were stationary at level I(0), while others were stationary at first difference I(1) [33, 34]. Notably, polynomial unit root tests yielded mixed results, with root values ranging between 0.98 and 0.03. Consequently, the variables exhibit different orders of integration. This implies that the models employed in the analysis may include both level-stationary and difference-stationary variables, highlighting the importance of conducting causality analyses while accounting for this heterogeneity [31, 32] (Table 4).

**Table 4:** Unit root test results

Variables	Level		Levin, Lin ve Chu	Breitung t-stat	IM, Pesaran and Shin W-stat	ADF	PP
SDGScore	Level	İnvidual Effects	0.2623	-	0.9583	0.4261	0.3383
		İnvidual Effects and Trends	0.0000*	0.8802	0.0000*	0.0074**	0.0382**
		None	1.0000	-	-	1.0000	1.0000
	1.diff.	İnvidual Effects	0.0000*	-	0.0000*	0.0000*	0.0000*
		İnvidual Effects and Trends	0.0000*	0.0586	0.0000*	0.0000*	0.0000*
		None	0.0000*	-	-	0.0000*	0.0000*
Matmort	Level	İnvidual Effects	0.0000*	-	0.1259	0.0242**	0.0000*
		İnvidual Effects and Trends	0.3079	0.9999	0.9408	0.6439	0.4761
		None	0.0000*	-	-	0.0000*	0.0000*
	1.diff.	İnvidual Effects	0.0379**	-	0.0124**	0.0036*	0.0000*
		İnvidual Effects and Trends	0.0019*	0.7246	0.0002*	0.0003*	0.0000*
		None	0.0000*	-	-	0.0004*	0.0000*
Neonat	Level	İnvidual Effects	0.0000*	-	0.0000*	0.0000*	0.0000*
		İnvidual Effects and Trends	0.0000*	0.9971	0.0000*	0.0000*	0.7622
		None	0.0000*	-	-	0.0001*	0.0000*
	1.diff.	İnvidual Effects	0.0000*	-	0.0011*	0.0001*	0.9598
		İnvidual Effects and Trends	0.0398**	0.2796	0.3800	0.0224**	0.9938
		None	0.0000*	-	-	0.0002*	0.0002*
U5Mort	Level	İnvidual Effects	0.0000*	-	0.0000*	0.0000*	0.0000*
		İnvidual Effects and Trends	0.0000*	0.9940	0.0000*	0.0000*	0.0000*
		None	0.0058**	-	-	0.0074**	0.0000*
	1.diff.	İnvidual Effects	0.0000*	-	0.0007*	0.0005*	0.0000*
		İnvidual Effects and Trends	0.0000*	0.9009	0.0033*	0.0006*	0.4919
		None	0.0000*	-	-	0.0000*	0.0000*
Edu	Level	İnvidual Effects	0.3464	-	0.0977***	0.0340**	0.0000*
		İnvidual Effects and Trends	0.1769	0.6922	0.0523**	0.0239**	0.0000*
		None	0.9410	-	-	0.9933	0.9991
	1.diff.	İnvidual Effects	0.0000*	-	0.0000*	0.0000*	0.0000*
		İnvidual Effects and Trends	0.0000*	0.0481**	0.0000*	0.0000*	0.0000*
		None	0.0000*	-	-	0.0000*	0.0000*
Hexp	Level	İnvidual Effects	0.0287**	-	0.4842	0.5829	0.9274
		İnvidual Effects and Trends	0.2794	0.1777	0.6749	0.7208	0.9979
		None	0.8055	-	-	0.9939	0.9983
	1.diff.	İnvidual Effects	0.0004*	-	0.0047**	0.0124**	0.0031*
		İnvidual Effects and Trends	0.0015*	0.1079	0.0268**	0.0481**	0.0164**
		None	0.0000*	-	-	0.0000*	0.0000*

Source: Prepared by the authors; *,**,***significance at %1,%5,%10 level.respectively

### Granger causality tests

The Granger causality test, developed by Granger (1969), is a statistical method used to determine whether the past values of one time series contain predictive information about another [35]. In other words, it tests whether changes in one variable precede and help forecast changes in another variable over time [35]. While it does not imply a true causal relationship, it provides important insights into temporal dynamics between variables [35]. In this study, the Granger causality test was applied to assess whether maternal and child mortality rates could statistically predict variations in SDG performance across the Turkic Republics.The results of the Granger causality analyses were presented in Table 5. The findings indicated that the SDG score had a significant causal effect on the under-five mortality rate (*p* =.017), while no significant effects were found on other variables (*p* >.05). Maternal mortality rate showed significant causality towards neonatal mortality rate (*p* =.008) and education (*p* =.099). Neonatal mortality rate significantly affected the SDG score (*p* =.022), maternal mortality rate (*p* =.056), and under-five mortality rate (*p* <.001). The under-five mortality rate exhibited a strong causal effect only on neonatal mortality rate (*p* <.001). Education did not show significant causality on other variables (*p* >.05). Health expenditure demonstrated a significant causal relationship only with the SDG score (*p* =.049). The tested models did not exhibit issues of serial correlation (LM test, *p* =.158) or heteroscedasticity (*p* =.144). The characteristic polynomial roots being less than one further supported the stability of the model. These results show that the SDG score was particularly affected by neonatal mortality and health expenditures in the short term, and that maternal and child health indicators interact strongly with each other. At the same time, these results highlighted the critical importance of neonatal and maternal health indicators in sustainable development performance and their interrelationships.

**Table 5:** Granger causality tests results

Hipotesis	Probability	Result
SDGScore ≠>Matmort	0.9481	Accepted
SDGScore ≠>Neonat	0.5179	Accepted
SDGScore ≠>U5Mort	0.0167**	**Rejected**
SDGScore ≠>Edu	0.5299	Accepted
SDGScore ≠>Hexp	0.6368	Accepted
Matmort ≠>SDGScore	0.6089	Accepted
Matmort ≠>Neonat	0.0084*	**Rejected**
Matmort ≠>U5Mort	0.2986	Accepted
Matmort ≠>Edu	0.0988***	**Rejected**
Matmort ≠>Hexp	0.6973	Accepted
Neonat ≠>SDGScore	0.0222**	**Rejected**
Neonat ≠>Matmort	0.0563***	**Rejected**
Neonat ≠>U5Mort	0.0006*	**Rejected**
Neonat ≠>Edu	0.9685	Accepted
Neonat ≠>Hexp	0.6139	Accepted
U5Mort ≠>SDGScore	0.1638	Accepted
U5Mort ≠>Matmort	0.2341	Accepted
U5Mort ≠>Neonat	0.0000*	**Rejected**
U5Mort ≠>Edu	0.5798	Accepted
U5Mort ≠>Hexp	0.8795	Accepted
Edu ≠>SDGScore	0.1196	Accepted
Edu ≠>Matmort	0.6125	Accepted
Edu ≠>U5Mort	0.6900	Accepted
Edu ≠>Neonat	0.1975	Accepted
Edu ≠>Hexp	0.5583	Accepted
Hexp ≠>SDGScore	0.0492**	**Rejected**
Hexp ≠>Matmort	0.9760	Accepted
Hexp ≠>u5mort	0.1761	Accepted
Hexp ≠>Neonat	0.4353	Accepted
Hexp ≠>Edu	0.9632	Accepted

Source:Prepared by the author

Roots of Characteristic Polynomial:0.981632 − 0.036782; Serial Correlation LM Tests:0.1584; Residual Heteroskedasticity Tests: 0.1440;*,**,*** significance at %1,%5,%10 level respectively

### Variance decomposition (Panel VAR, Monte-Carlo = 100000)

In this study, variance decomposition analysis was employed within the panel VAR framework to assess the relative contribution of maternal and child mortality to the fluctuations in SDG performance. This technique allows for the quantification of the extent to which shocks in health indicators explain variations in sustainable development outcomes over time. As such, it provides valuable insight into the dynamic interdependencies among the variables under investigation [36–38]. Variance decomposition analysis was performed to evaluate the relative contribution of independent variables to the variance of the SDG score from 1 to 10 periods ahead. In the first period, 100% of the variance in SDG score was explained by its own lagged values. However, over time, the influence of maternal mortality rate increased from 0% in period 1 to 6.89% by period 10, under-five mortality rate rose from 0% to 8.65%, neonatal mortality rate contributed between 0% and approximately 2.27%, education increased from 0% to 1.08%, and health expenditure accounted for up to 6.68% of the variance by period 10. These findings suggested that health and education factors exerted a dynamic and cumulative influence on sustainable development outcomes, demonstrating the model’s capacity to capture temporal interactions effectively. The results showed that, initially, the SDG score was largely determined by its own past values, while over time the explanatory variables increasingly contributed to its variability. In summary, changes in SDG scores were primarily driven by maternal and child health indicators, with education and health expenditures becoming progressively more influential as time progressed.

## Discussion and recommendation

The relationship between SDG score and maternal, neonatal, and child mortality constitutes a central theme in global health policy and research. These health indicators are particularly significant within the framework of SDG 3, which aims to “ensure healthy lives and promote well-being for all at all ages.” SDG 3.1 specifically targets the reduction of the global maternal mortality ratio to fewer than 70 per 100,000 live births by 2030. Maternal health is closely intertwined with other indicators such as neonatal and under-five child mortality, as well as broader access to healthcare services, including skilled birth attendance and quality postnatal care. However, disparities persisted—especially in low-income countries—where approximately 95% of maternal deaths arose from preventable causes such as hemorrhage and infection. In this context, strengthening healthcare infrastructure and addressing gender inequalities are fundamental prerequisites for achieving these goals [14, 39–42].

This study employed a panel data analysis covering the period 2000–2020 across six Turkic Republics to investigate the main determinants of SDG performance. The results provided valuable evidence on how maternal and child health metrics, levels of educational attainment, and healthcare spending influence SDG performance.

The analysis revealed a negative and statistically significant impact of maternal mortality on SDG performance, corroborating earlier studies. This finding is consistent with Filmer and Pritchett (1999), who highlight the critical importance of healthcare systems and maternal health in driving overall development outcomes [43]. Moreover, it has been proposed that elements such as the availability of antenatal care, accessibility of health services, and gender equality may exert an indirect effect on SDG outcomes by shaping maternal mortality levels [44, 45].

The fixed effects panel model indicated that under-five mortality (u5mort) had a statistically significant negative impact on SDG scores. This outcome is consistent with prior studies. For instance, reports by UNICEF (2019) and WHO (2020) highlighted that elevated child mortality not only undermines health outcomes but also adversely affects other SDG dimensions, including education, economic development, and social equity [45–47]. Similarly, Sachs (2015) emphasized that child health plays a pivotal role in sustainable development and is closely linked to overall national development levels [48]. The analysis further revealed that education exerted a statistically significant positive influence on SDG outcomes. This finding aligns with the work of Barro and Lee (2013) and Hanushek and Woessmann (2008), who highlighted the strong causal relationship between educational attainment and development. Higher levels of education not only enhance public awareness but also improve access to healthcare services and promote the adoption of more sustainable practices at the individual level [49, 50]. Conversely, neither health expenditure nor neonatal mortality exhibited statistically significant effects within the model. This phenomenon has been widely discussed in the literature. For example, Deaton (2013) suggested that in contexts where the efficiency of health spending is low, the impact of healthcare expenditures on development outcomes may be constrained [51]. These results imply that, beyond the overall level of health expenditure, the efficiency of resource utilization and the quality of healthcare services play a more decisive role in advancing development. The analysis further identified both unidirectional and bidirectional statistically significant causal relationships among the variables. Of particular interest was the reciprocal link between SDG scores and child health indicators, suggesting that improvements in sustainable development and health outcomes mutually reinforce one another. This conclusion aligns with the viewpoints of Sachs (2015) and UNICEF (2019), who emphasize that child health should be considered not only as a development outcome but also as a key driver influencing broader developmental pathways [46, 48]. Moreover, the causal links observed from maternal mortality to both education and neonatal mortality rates highlighted that health indicators were influenced not only by healthcare systems but also by broader social determinants such as education. This was supported by OECD (2022) reports, which underlined the strong connection between women’s health and educational attainment [44].

Higher maternal and under-five mortality rates were linked to lower SDG scores, while education positively influenced development outcomes (Table 2). Stationary variables and variance decomposition results further indicated that these health and education factors had enduring effects, highlighting a persistent long-term relationship among key development indicators (Tables 4, 5 and 6) [43, 50]. Recent studies highlighted the reciprocal impacts of technological advancements and environmental risks on social change and human well-being [52]. Particularly in the case of South Asia, the interaction between these two factors shaped the multidimensional nature of sustainable development. These findings corroborated the importance of the interconnections among health, education, and economic indicators identified in our study and emphasized the necessity of multidimensional and integrated policy approaches to achieve the SDG targets [53, 54]. Thus, sustainable development is underpinned by deep structural linkages across social, economic, and health domains.

**Table 6:** Variance decomposition analysis results of SDGScore and health indicators*

	SDGScore	Matmort	Neonat	U5Mort	Edu	Hexp
1	100.00	0.00	0.00	0.00	0.00	0.00
2	93.99	1.42	3.12	0.39	0.03	1.05
3	92.07	2.31	3.52	1.22	0.08	0.77
4	90.14	2.70	3.39	2.65	0.42	0.69
5	88.17	3.20	2.98	4.20	0.62	0.83
6	85.79	3.92	2.62	5.68	0.69	1.28
7	83.16	4.55	2.42	6.93	0.80	2.13
8	80.23	5.28	2.34	7.83	0.91	3.39
9	77.28	6.08	2.32	8.39	0.99	4.94
10	74.43	6.89	2.27	8.65	1.08	6.69

Source: Prepared by the authors

*Estimated under 100.000 monte carlo simulation

This study shed light on the interplay between maternal and child health, education, and health expenditures in relation to the SDG by conducting a panel data analysis across six Turkic Republics. The results underscore the pivotal role of health and education indicators in driving the development process, revealing the presence of long-term structural interconnections among these variables. Accordingly, the following policy implications are suggested (i) Policymakers should strengthen healthcare systems targeting maternal and child health and expand access to education to support the achievement of SDG targets. In this context, enhancing women’s educational attainment and making their labor visible [55] play a crucial role in ensuring sustainable development; (ii) Elevating education levels is critical for sustainable development, with particular attention to promoting girls’ access to education; (iii) Health expenditures should be allocated efficiently and effectively, ensuring that resources directly improve health outcomes; (iv) Policies promoting gender equality need to be reinforced to expand women’s access to healthcare services; (v) Greater coordination among health, education, and economic policies is essential to facilitate the achievement of SDG targets.

### Conclusion and limitation

This research underscores the substantial influence of maternal and child health indicators, alongside educational attainment, on the advancement of sustainable development within the Turkic Republics. The findings indicate that elevated maternal and under-five mortality rates hinder SDG performance, while higher levels of education exert a positive effect. The presence of long-term associations and causal relationships highlights the necessity of continuous investment in both health and education sectors to foster sustainable development across the region. Accordingly, policymakers are encouraged to strengthen healthcare systems targeting mothers and children and to expand access to education to promote SDG achievements.

The study provides valuable insights into the interplay between health indicators and sustainable development within the selected Turkic Republics; however, certain limitations should be acknowledged. First, historical data availability varied across countries, especially during the early years of the 2000–2020 period. Second, the analysis covered only six Turkic Republics (Azerbaijan, Kazakhstan, Kyrgyzstan, Tajikistan, Turkey, and Uzbekistan), which restricts the generalizability of the findings to the broader region. Third, structural factors such as environmental sustainability, governance quality, and income distribution were not included in the model. This exclusion was intentional, aiming to maintain model simplicity and avoid multicollinearity issues.

Advanced econometric methods, including causality tests, were employed to ensure the robustness of the analysis. Potential challenges, such as structural breaks, external shocks, and unobserved heterogeneity, were addressed to a considerable extent. Nevertheless, interpretations of long-term relationships should be made cautiously, considering contextual differences and policy variations across countries.

## Data Availability

Data were obtained from https://www.sdgindex.org and https://www.worldbank.org.

## Abbreviations

SDGs: Sustainable Development Goals
FEVD: Forecast Error Variance Decomposition
OLS: Ordinary Least Squares
GDP: Gross Domestic Product
VIF: Variance Inflation Factors
LM test: Lagrange Multiplier test for serial correlation
Levin–Lin–Chu: Levin–Lin–Chu unit root test
Breitung: Breitung unit root test
Im–Pesaran–Shin: Im–Pesaran–Shin unit root test
ADF-Fisher: Augmented Dickey-Fuller Fisher test
PP-Fisher: Phillips-Perron Fisher test
LR: Likelihood Ratio
FPE: Final Prediction Error
AIC: Akaike Information Criterion
SC: Schwarz Criterion (Bayesian Information Criterion)
HQ: Hannan–Quinn Criterion
AR(1): First-order Autoregressive process
Breusch–Pagan LM: Breusch–Pagan Lagrange Multiplier test
Pesaran scaled LM: Pesaran scaled Lagrange Multiplier test
Bias-corrected LM: Bias-corrected Lagrange Multiplier test
Pesaran CD: Pesaran Cross-sectional Dependence test
UNICEF: United Nations International Children’s Emergency Fund
OECD: Organisation for Economic Co-operation and Development
